# Multimodal Assessment of Ocular Parameters in Patients with Severe Obstructive Sleep Apnea with Emphasis on Retinal Structural Changes

**DOI:** 10.3390/life15081307

**Published:** 2025-08-18

**Authors:** Anita Pusic Sesar, Anja Cehajic, Antonela Geber, Mia Zoric Geber, Ivan Cavar, Antonio Sesar

**Affiliations:** 1 Department of Ophthalmology, School of Medicine, University of Mostar, 88 000 Mostar, Bosnia and Herzegovina; pusicanita@gmail.com (A.P.S.); antoniosesar@yahoo.com (A.S.); 2 Department of Anesthesia and Intensive Care, University Hospital Center Mostar, 88 000 Mostar, Bosnia and Herzegovina; anjacehajic42@gmail.com; 3 Department of Ophthalmology, University Hospital Center Zagreb, 10000 Zagreb, Croatia; 4 Department of Ophthalmology, Sestre Milosrdnice University Hospital Center, 10000 Zagreb, Croatia; miazoricgeber@gmail.com; 5 Department of Physiology and Immunology, School of Medicine, University of Mostar, 88 000 Mostar, Bosnia and Herzegovina

**Keywords:** obstructive sleep apnea, glaucoma, intraocular pressure, central corneal thickness, optical coherence tomography, retinal nerve fiber layer, ganglion cell layer

## Abstract

Background: Obstructive sleep apnea (OSA) is increasingly recognized as a potential risk factor for glaucoma due to its association with intermittent hypoxia and vascular dysregulation. The aim of this study was to investigate early glaucomatous changes in the eyes of patients with OSA. Methods: This case–control study included 25 patients with OSA and 25 age- and sex-matched healthy controls. According to the STOP-Bang Questionnaire, patients with an intermediate or severe risk of OSA underwent polysomnography. Based on the apnea-hypopnea index, only patients with severe OSA were included in the study group. All participants underwent a full ophthalmological examination, with measurements of the retinal nerve fiber layer (RNFL) and ganglion cell layer (GCL) thickness performed using optical coherence tomography (OCT). Results: Statistical comparisons revealed that intraocular pressure (IOP) was slightly elevated, while central corneal thickness (CCT) was slightly reduced in patients with OSA compared to healthy controls. OCT measurements showed an overall reduction in both RNFL and GCL thicknesses in the OSA group. Among these findings, only the differences in average and minimum GCL thickness in the left eye reached statistical significance. Conclusions: The results of our study indicate significant thinning of the GCL in patients with OSA, suggesting possible early glaucomatous changes and a potential link between OSA and glaucoma. Given the increasing prevalence of OSA, further large-scale, long-term studies are needed to better understand this relationship and its clinical implications.

## 1. Introduction

Obstructive sleep apnea (OSA) is one of the most common sleep-related breathing disorders, characterized by episodes of partial or complete obstruction of the upper airway. The key features of OSA are recurrent pauses in breathing, which lead to intermittent hypoxia and hypercapnia, accompanied by oxygen desaturation and arousal during sleep [1,2]. OSA is a growing public health concern affecting nearly one billion people worldwide and is considered an independent cardiovascular risk factor, commonly associated with obesity, insulin resistance, hypertension, stroke, coronary artery disease, heart failure, and arrhythmias [3]. Major risk factors for the development of OSA include older age, male sex, and obesity. Additional contributing factors may include genetic predisposition, ethnic background, menopause, and unhealthy lifestyle habits such as smoking and alcohol consumption [1,2]. Intermittent hypoxia, as a key mediator in the pathogenesis of disorders associated with OSA, triggers sympathetic activation, low-grade inflammation, and oxidative stress, all of which contribute to tissue damage [4]. The diagnostic gold standard for OSA is overnight polysomnography, conducted either in a sleep laboratory or under ambulatory conditions, to record respiratory events during sleep and calculate the apnea–hypopnea index (AHI) [3,5]. Continuous positive airway pressure (CPAP) is the most effective treatment, shown to improve quality of life and sleep parameters in patients with OSA. Other treatment modalities include weight loss through diet and exercise, oral appliance therapy, and surgical interventions to correct anatomical airway obstructions [5].

OSA has been associated with a range of ocular diseases, including glaucoma, optic disc edema, non-arteritic anterior ischemic optic neuropathy, central serous chorioretinopathy, diabetic retinopathy, floppy eyelid syndrome, keratoconus, retinal vein occlusion, and age-related macular degeneration, although findings across studies have been somewhat inconsistent [6,7,8,9,10]. Furthermore, individual reports suggest that the treatment of OSA may have beneficial effects on eye diseases [6,7,8]. Glaucoma is a progressive optic neuropathy characterized by degeneration and loss of retinal ganglion cells, resulting in optic disc cupping, visual field defects, and thinning of the retinal nerve fiber layer (RNFL) and ganglion cell layer (GCL), all of which contribute to irreversible visual loss. As such, it is one of the leading causes of irreversible blindness worldwide, affecting more than 70 million people [11,12].

The literature provides evidence that patients with OSA, especially those with severe disease, have a higher prevalence of glaucoma, and vice versa: patients with glaucoma, particularly normal tension glaucoma (NTG), also have a significant risk of developing sleep disorders. The considered pathophysiological basis of glaucomatous damage in patients with OSA includes hypoxia, vascular dysregulation, and elevated intraocular pressure (IOP) [13,14]. Several studies have reported a significant association between OSA and elevated IOP, with indicated higher IOP values in patients with moderate-to-severe OSA [15,16]. In contrast, the results of some studies have not shown significant differences in IOP values between OSA patients and control subjects [17,18]. Analyses of central corneal thickness (CCT) in individuals with OSA show no significant differences or may reveal slightly lower values [15,18]. Furthermore, numerous studies have reported structural changes in the optic disc and retina in patients with OSA, such as thinning of the RNFL and GCL, further reinforcing the potential association with glaucoma [19,20,21,22,23]. However, some authors have found no significant link between the two conditions or consider the association to be coincidental [24,25,26,27].

Despite numerous studies describing ocular changes in patients with OSA, the results remain inconclusive and lack comprehensiveness. Therefore, the aim of our study was to compare IOP, CCT, and optical coherence tomography (OCT) parameters, including RNFL and GCL thickness, between patients with OSA and healthy controls.

## 2. Materials and Methods

### 2.1. Study Design and Participants

This observational, case–control study was conducted from June 2024 to February 2025 at the Eye Clinic of the University Hospital Center Mostar and the Sleep Medicine Center of the University of Mostar School of Medicine, Bosnia and Herzegovina. A total of 50 subjects, comprising 34 men and 16 women and aged between 21 and 60 years, were recruited into the study. The mean age of the participants was 45.40 ± 9.79 years. Of the total sample, 28 individuals were emmetropic, 16 had myopia, and 6 had hypermetropia. The mean spherical equivalent for all participants was −0.36 ± 0.79 diopters; for patients with OSA, it was −0.30 ± 0.77 diopters, while for control subjects, it was −0.36 ± 0.81 diopters. The study group consisted of 25 patients with severe OSA without prior diagnosed ophthalmic conditions, while the control group included 25 healthy individuals who were matched with OSA patients according to sex, age (±2 years), and refractive status. The exclusion criteria were refractive errors greater than ±3.00 diopters, ocular surgeries or laser treatments, inflammatory and degenerative eye diseases, and glaucoma. Additional exclusion criteria for the control group included an intermediate or high risk of OSA development according to the STOP-Bang Questionnaire [28].

The sole purpose of the study was to collect data for scientific research. All participants provided informed consent before the research began, ensuring that they understood the procedures, risks, benefits, and purpose of the research. The consent form was provided in Croatian, enabling it to be accessible to all participants. Participants were informed that they could withdraw from the study at any time without providing a specific reason, and they were guaranteed full anonymity and protection of their personal data. Access to participants’ medical records was limited to the researchers involved in the study and members of the Ethics Committee. Ethical approval was obtained from the Ethics Committee of the School of Medicine, University of Mostar (Approval No. 01-1-288/24), which was in accordance with the ethical standards set by the Declaration of Helsinki and the Law on the Rights, Obligations, and Responsibilities of Patients of Bosnia and Herzegovina.

### 2.2. Data Collection

The risk of OSA was assessed using the STOP-Bang Questionnaire, presented in Table 1 [28].

Participants with an intermediate or high risk of OSA underwent polysomnography, which was conducted utilizing a 12-channel, 22-connector Respironics Alice 6LE device (Philips Respironics, Eindhoven, The Netherlands). This device recorded airflow, oxygen saturation, respiratory effort, cardiac electrical and skeletal muscle activity, brain waves, and eye movements. The AHI was employed to define OSA severity, measured as the total number of apneas and hypopneas per hour. The AHI is classified as follows: <5/h—no OSA, 5–15/h—mild OSA, 15–30/h—moderate OSA, and >30/h—severe OSA [29]. Only patients with severe OSA were included in the study group.

All subjects underwent a complete ophthalmological examination, including an assessment of best-corrected visual acuity using a Precision Vision 2.5-Metre ETDRS LogMAR Eye-Test (Health and Care, London, UK), measurement of IOP with a Goldmann applanation tonometer and the CCT by pachymetry (EchoScan US-1800, NIDEK Co., Ltd., Gamagori, Japan), slit-lamp biomicroscopic examination of the anterior eye segment (ZEISS SL 800 Slit lamp, Carl Zeiss Meditec AG, Jena, Germany), fundus examination in mydriasis utilizing a 90D Volk non-contact lens, and an evaluation of RNFL and GCL thickness with spectral-domain, high-definition OCT (SD-OCT; Carl Zeiss Meditec, Inc., Cirrus 5000, Dublin, CA, USA).

### 2.3. Statistical Analysis

Data were analyzed using IBM SPSS software (version 25.0; IBM Corp., Chicago, IL, USA) and Microsoft Excel (Office 2021, Microsoft Corporation, Redmond, WA, USA). The Shapiro–Wilk test for normality indicated that the data distribution was normal; therefore, the data are presented as means and standard deviations. Differences between groups were assessed by utilizing the Student’s *t*-test. Statistical significance was set at *p* < 0.05.

## 3. Results

### 3.1. Intraocular Pressure

**Figure 1:**
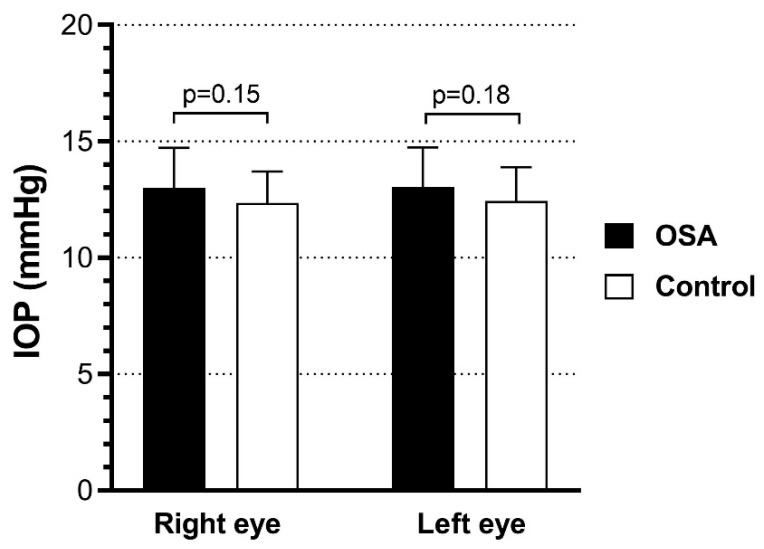
IOP values in OSA patients and control subjects.

Figure 1 shows the IOP values for both eyes in the study and control groups. IOP values (mm Hg) were 13.00 ± 1.73 (right eye) and 13.04 ± 1.70 (left eye) in OSA patients and 12.36 ± 1.35 (right eye) and 12.44 ± 1.45 (left eye) in control subjects. Statistical comparison indicated that IOP was higher in patients with OSA than in control subjects, although the difference was not statistically significant (*p* = 0.152 for the right eye and *p* = 0.184 for the left eye).

### 3.2. Central Corneal Thickness

**Figure 2:**
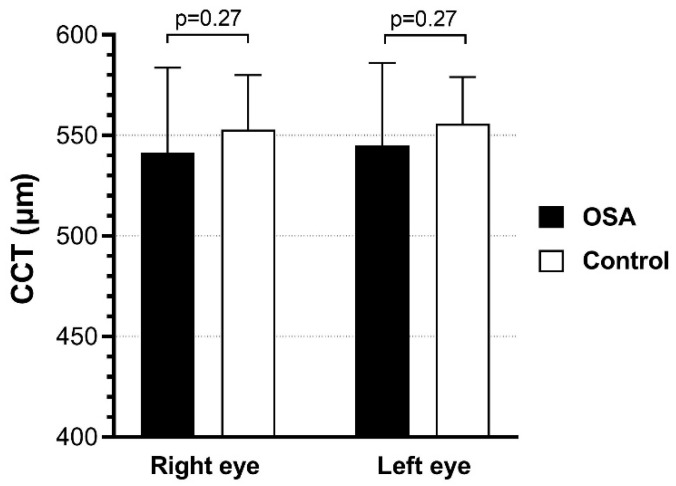
CCT values in OSA patients and control subjects.

Pachymetry measurements showed that CCT values (μm) were 541.56 ± 42.14 (right eye) and 545.16 ± 40.90 (left eye) in OSA patients, while they were 552.84 ± 27.31 (right eye) and 555.68 ± 23.33 (left eye) in the control group. Patients with OSA had lower CCT values compared to control subjects, although the difference was not significant for either the right (*p* = 0.267) or left eye (*p* = 0.270) (Figure 2).

### 3.3. Thickness of Retinal Layers

Table 2 shows that RNFL thickness (μm) in the right eye was slightly reduced in patients with OSA compared to controls across all measurements: average (*p* = 0.801), superior (*p* = 0.778), inferior (*p* = 0.704), nasal (*p* = 0.795), and temporal (*p* = 0.414) RNFL. In the left eye, RNFL thickness was also slightly reduced in patients with OSA: average (*p* = 0.564), superior (*p* = 0.806), inferior (*p* = 0.864), nasal (*p* = 0.739), and temporal (*p* = 0.403) RNFL.

**Figure 3:**
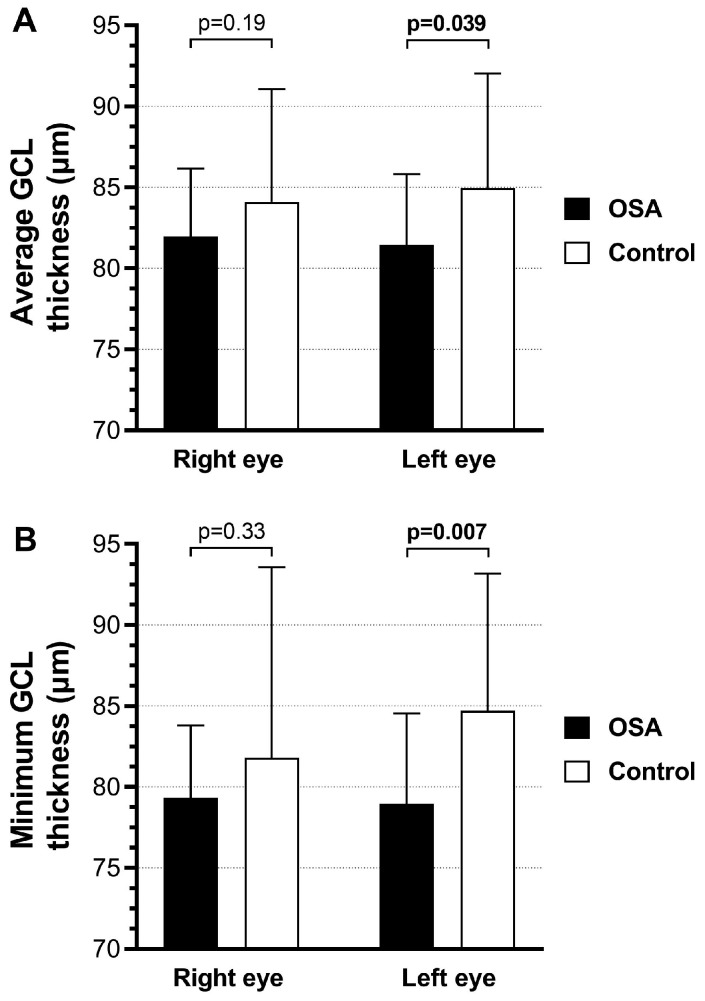
GCL thickness in study participants. (**A**) Average GCL thickness; (**B**) Minimum GCL thickness.

Average and minimum GCL thickness (μm) in both study groups are shown in Figure 3. Average GCL thickness values were 81.96 ± 4.20 (right eye) and 81.44 ± 4.38 (left eye) in OSA patients and 84.12 ± 6.95 (right eye) and 84.96 ± 7.06 (left eye) in control subjects. Patients with OSA had decreased values of average GCL thickness compared to the control group, whereby that difference was not significant for the comparison of right eyes (*p* = 0.19), but it was for the comparison of left eyes (*p* = 0.039) (Figure 3A).

Minimum GCL thickness values were 79.32 ± 4.48 (right eye) and 78.96 ± 5.60 (left eye) in OSA patients and 81.80 ± 11.77 (right eye) and 84.72 ± 8.46 (left eye) in control subjects. OSA patients had decreased values of minimum GCL thickness compared to the control subjects, whereby that difference was not significant for the comparison of right eyes (*p* = 0.33), but it was for the comparison of left eyes (*p* = 0.007) (Figure 3B).

## 4. Discussion

OSA is currently a global health issue, characterized by recurrent episodes of partial or complete airway obstruction during sleep. There are numerous publications reporting on a potential relationship between OSA and different eye diseases, among which glaucoma has been the most prominent subject of debate in recent years [6,30,31]. Previously, analyses about the connection between OSA and glaucoma reported a relatively high prevalence of glaucoma, ranging from 5.7% to 27% in patients with OSA [32]. A recent systematic review and meta-analysis estimated that OSA is associated with up to a 1.5-fold increase in the odds of developing glaucoma [30,31].

Our results showed that IOP values in OSA patients were slightly higher in comparison to controls without statistical significance. Other researchers have established that IOP is significantly higher in OSA patients and that OSA severity correlates with IOP [15,16]. In our study, patients with OSA showed slightly lower CCT values compared to controls. However, this difference was not statistically significant. According to current clinical guidelines, including those issued by the European Glaucoma Society, IOP should not be adjusted based on individual CCT measurements [11]. CCT is considered an independent risk factor that may influence the assessment of glaucoma risk, especially in eyes with thinner corneas. IOP values should therefore be interpreted as measured, without modification based on CCT. Conversely, Adam et al. reported that there was no difference between the OSA and control groups regarding IOP and central corneal thickness [17]. Oxidative stress may contribute to trabecular meshwork damage, leading to IOP elevation, although the exact pathophysiology remains unclear [33]. Furthermore, Tsang et al. have confirmed that patients with moderate to severe OSA may exhibit significant visual field defects and an increased prevalence of glaucomatous changes at the optic nerve head, even in the absence of elevated intraocular pressure [34]. The latest studies suggest that higher STOP-Bang scores, which indicate a more severe form of OSA, are associated with a higher incidence of glaucoma and greater values of intraocular pressure [35].

Pachymetry in OSA patients had lower values when compared to control subjects, but these were also without statistical significance. Studies that analyzed corneal parameters in OSA patients showed that there are no differences in corneal thickness between severe OSA patients and healthy controls [36,37]. On the contrary, Bojarun et al. noted a thinner cornea in OSA patients, suggesting that the severity of hypoxemia can reduce corneal thickness [38].

The results of our study revealed that RNFL thickness in OSA patients was slightly reduced in both eyes. Several works have reported a thinning RNFL in patients with OSA and a negative correlation between the AHI and RNFL thickness [20,22,39,40,41]. Acute ischemia may initially cause neuronal swelling, followed by chronic degeneration and thinning in later phases [20]. Due to hypoxemia, there is an increased level of vasoconstrictor endothelin production and an impaired vasodilatory response. An imbalance between nitric oxide and endothelin, as well as disturbed ocular perfusion, caused by hypoxia and vascular dysregulation, may be the reason for RNFL thinning [42].

The analysis of average and minimum GCL thickness showed thinning in both eyes, while statistically significant differences were only identified in the left eye. Kara et al. also confirmed the thinning of average and minimum GCL thickness in patients with severe OSA compared to controls [43]. In contrast, Ferrandez et al. did not confirm statistically significant GCL thinning in their study [44]. Some authors suggest that reduced GCL thickness in the absence of RNFL thinning may indicate that GCL analysis could be a more sensitive method for detecting early glaucomatous damage [43].

Additionally, it was found that OSA severity was negatively correlated with the rate of GCL thickness in the NTG subgroup [45]. The evaluation of patients, which included overnight polysomnography, reported a higher prevalence of OSA in NTG patients. The authors conclude that OSA should be considered a significant risk factor for NTG and recommend taking an accurate sleep history, as well as referring NTG patients for polysomnography testing and nasal continuous positive airway pressure therapy [46]. In addition, patients with OSA were 6.72 times as likely to develop NTG and two times as likely to develop primary open-angle glaucoma (POAG) compared to patients without OSA [47].

On the contrary, a study that analyzed the risk of OSA in open-angle glaucoma patients using the STOP-BANG Questionnaire found no evidence that glaucoma patients are more likely to have OSA or more severe OSA than others [48]. Wozniak et al. identified a relatively high prevalence of OSA in POAG patients, but it was not significantly higher than in those without glaucoma. Therefore, this result does not support screening for OSA in patients with POAG [49].

The observed increased risk of glaucoma in patients with OSA and vice versa, the heightened risk of OSA in patients with glaucoma, strongly suggest an association between these two diseases [50]. In addition, both share similar pathophysiological mechanisms, such as hypoxia, oxidative stress, and inflammation, with the expected increased risk of their development in older age [47].

As with any clinical study, several limitations must be considered. The relatively small sample size, the inclusion of only severe OSA cases, and the case–control design may limit the generalizability of the findings and prevent conclusions regarding causality. Although certain parameters, such as IOP, CCT, and RNFL thickness, showed trends toward intergroup differences, these did not reach statistical significance. This outcome may be partly explained by the limited sample size and reduced statistical power, which can obscure subtle yet clinically meaningful differences. Similar observations have been documented in previous studies with sample sizes comparable to ours, in which structural retinal changes in patients with OSA were noted but did not consistently reach statistical significance [24,42,51]. Variability in findings across studies may be attributed to differences in study design, population characteristics, sample size, inclusion criteria, imaging protocols, and the presence of comorbid conditions. An additional limitation of the present study is the lack of longitudinal follow-up, which prevents evaluation of disease progression over time. Moreover, comprehensive glaucoma diagnostics, such as visual field testing, were not conducted, making it difficult to confirm whether the observed thinning of the GCL is specific to glaucomatous damage. Another limitation is the absence of data on CPAP therapy status. Since CPAP has been shown to influence ocular parameters [52,53], failure to account for treatment effects could confound the observed associations. Despite these limitations, the study provides meaningful insights that contribute to the understanding of the relationship between OSA and retinal structural changes.

## 5. Conclusions

The results of our study demonstrate significant thinning of the GCL in patients with OSA, suggesting the presence of early glaucomatous changes and a potential association between OSA and glaucoma. Additionally, a slight elevation in IOP, along with mild reductions in CCT and RNFL thickness, was observed in the OSA group compared to healthy controls, although these differences did not reach statistical significance. These subtle yet consistent changes may reflect early retinal involvement driven by chronic intermittent hypoxia and vascular dysregulation associated with OSA. The findings support the clinical value of OCT in the early detection of retinal alterations in patients with OSA and underscore the importance of further research to clarify the impact of OSA presence, duration, and severity on progressive retinal damage. Therefore, future studies with larger and more heterogeneous cohorts, combined with longitudinal follow-up, are warranted to elucidate the long-term ophthalmologic consequences of OSA.

## Figures and Tables

**Table 1:** STOP-Bang Questionnaire.

STOP		
Do you SNORE loudly (louder than talking or loud enough to be heard through closed doors)?	YES	NO
Do you often feel TIRED, fatigued, or sleepy during daytime?	YES	NO
Has anyone OBSERVED you stop breathing during your sleep?	YES	NO
Do you have or are you being treated for high blood PRESSURE?	YES	NO
**Bang**		
BMI more than 35 kg/m^2^?	YES	NO
AGE over 50 years old?	YES	NO
NECK circumference > 16 inches (40 cm)?	YES	NO
GENDER: Male?	YES	NO
**Total score**		
High risk of OSA: Yes 5–8 Intermediate risk of OSA: Yes 3–4 Low risk of OSA: Yes 0–2		

**Table 2:** RNFL thickness in the study participants.

RNFL Thickness (μm)	OSA Patients (Mean ± SD)	Control Subjects (Mean ± SD)	t	*p*
**Right eye**				
Average	95.00 ± 9.566	95.68 ± 9.433	0.253	0.801
Superior	114.60 ± 15.885	115.84 ± 14.974	0.284	0.778
Inferior	121.08 ± 13.534	122.64 ± 15.253	0.383	0.704
Nasal	72.72 ± 12.468	73.56 ± 10.182	0.261	0.795
Temporal	66.24 ± 7.758	68.24 ± 9.338	0.824	0.414
**Left eye**				
Average	92.96 ± 9.062	94.48 ± 9.426	0.581	0.564
Superior	118.64 ± 19.666	119.88 ± 15.619	0.247	0.806
Inferior	118.88 ± 13.639	128.20 ± 14.303	0.172	0.864
Nasal	70.04 ± 9.312	71.04 ± 11.639	0.335	0.739
Temporal	63.76 ± 7.423	65.60 ± 7.974	0.844	0.403

## Data Availability

The data supporting the findings of this study are not publicly available due to the presence of personal information and ethical considerations.

## Abbreviations

The following abbreviations are used in this manuscript:

AHI	Apnea–hypopnea index
BMI	Body mass index
CCT	Central corneal thickness
CPAP	Continuous positive airway pressure
GCL	Ganglion cell layer
IOP	Intraocular pressure
NTG	Normal tension glaucoma
OCT	Optical coherence tomography
OSA	Obstructive sleep apnea
POAG	Primary open-angle glaucoma
RNFL	Retinal nerve fiber layer
SD	Standard deviation
SD-OCT	Spectral-domain optical coherence tomography
